# Prospective Comparative Observational Study of Safety and Efficacy of Topical Ozone Gas Therapy in Healing of Diabetic Foot Ulcers versus Only Conventional Wound Management

**DOI:** 10.1055/s-0041-1731447

**Published:** 2021-09-14

**Authors:** Suchin Dhamnaskar, Nishant Gobbur, Mandar Koranne, Dhaval Vasa

**Affiliations:** 1 Department of Surgery, Seth G.S. Medical College and K.E.M. Hospital, Mumbai, Maharashtra, India

**Keywords:** diabetic foot ulcers, topical ozone gas therapy, wound healing

## Abstract

The prevalence of diabetic foot ulcers (DFUs) in India is 11.6%. DFU accounts for major cost expenditure, morbidity, and mortality. 1 Ozone gas has antimicrobial and antioxidant properties. We studied efficacy of topical ozone gas therapy in promoting healing of DFU. This is an observational comparative cohort study, *n* = 160, There were two groups of patients namely: those who received Conventional wound management alone C and those who received topical ozone therapy in addition to conventional wound management O + C therapy groups (81 each). Study group, i.e., O + C received five alternate day sessions of ozone therapy by bagging method for 30 minutes each session. Both groups were observed for 30 days for wound healing parameters like reduction of wound surface area, wound diameter, presence and character of discharge, granulation tissue, healing wound edges, microbial negativity, and requirement of revision (re-debridement and/or amputation) surgery. Mean baseline ulcer surface area is 17.43 ± 8.6 cm ^2^ for C and 17.87 ± 9.2 cm ^2^ (range 1–50 cm ^2^ ) in O + C group. Percentage change in ulcer surface after 21 days in O + C group is 32.37% compared with 17.15% in C group, which is statistically significant ( *p* = 0.01). Rates of microbial negativity and ulcer healing were significantly faster in ozone group. There was a statistically significant decrease in hospital stay, number of revision surgeries required, and mortality in ozone group. Topical ozone gas was well tolerated. Our study supports the efficacy of ozone therapy in DFU healing and reduction in the chances of infection and revision (re-debridement and/or amputation) surgery. More research is needed for dose, duration, and exposure time standardization.

One in four diabetes mellitus (DM) patients are reported to develop diabetic foot ulcer (DFU). 1 The prevalence of DFU in India is 11.6% (95% CI: 6.4–16.8%). The above data shows that DFU is a very common, complicated, and costly problem that draws researchers' interest to find effective means to prevent or treat it and help its better healing. Ozone is a gas made of three atoms of oxygen with a cyclic structure and can be applied to treat many diseases due to its antioxidant and anti-bacterial properties. For instance, it can be used in the treatment of chronic infections caused especially by antibiotic-resistant pathogens. Recently, the beneficial effects have been found of treating a vascular ulcer with ozone. Moreover, ozone administration can induce tolerance of oxidative stress and prevent damage mediated by free radical. 2

## Aims and Objectives

### Aim

The aim of the study is to assess the efficacy and safety of topical ozone gas therapy for healing of DFUs.

### Objectives

1. Primary objective:
  - To assess the rate of wound healing in diabetic ulcers.
  - To assess the adverse effects of ozone gas therapy.
2. Secondary objective:
  - To assess the rate of achieving local microbial negativity of wound swabs.
  - To assess the reduction in hospital stay.
  - To assess the rate of formation of healthy granulation tissue.

## Material and Methods

*Study design* : Prospective comparative cohort observational study.
*Study site* : Study conducted in tertiary care teaching hospital at developing country.
*Study duration* : Until the estimated sample size is achieved (81 in each arm).

### Inclusion Criteria

1. All the patients, men or women, with either type-1 or type-2 DM with a Wagner classification stages 2, 3 or post debridement stage 4 without venous insufficiency or lymphatic obstruction and without spreading cellulitis.
2. Age above 18 years.

### Exclusion Criteria

Wound size of >50 cm ^2^ , gangrenous foot ulcers, active osteomyelitis, history of hypo or hyperthyroidism, hemoglobin A1c (HbA1c) >10%, ankle-brachial index <0.8, hemoglobin level <8 g%, serum creatinine level >2.5 mg/dL or patient on dialysis, serum albumin level <2 g/dL, liver function tests (alanine transaminases, aspartate transaminases) elevated to more than three times the upper normal limit, patients with collagen vascular diseases, patients on steroid therapy, immunosuppressed host, known cancer patient, pregnant females, and post-partum females.

### Withdrawal Criteria

The criteria include patients withdrawing consent for further participation in due course of study at any point of time due any of the adverse effects like pungent smell of ozone gas, etc.

### Sample Size Calculation

*N* = ( *Z* α _/2_ + *Z* β) ^2^ /( *p* 1 − *p* 2) ^2^ × *P* × (1 − *P* )

*N* = size per group;

*p* 1 = % of ulcers healed in Ozone plus C Group 24% 3

*p* 2 = % of ulcers healed in C Group 12% 3

*P* = pooled prevalence *p* 1 + *p* 2/2 = 24 + 12/2 = 18 *p* = 18

*Zα* /2 = *Z* 0.1/2 = *Z* 0.05 = 1.96 — From *Z* table at type I error of 10%

*Zβ* = *Z* 0. 20 = 0.842—at 80% power

Sample size 81 patients in each group.

## Procedure

Study was conducted after institutional ethical approval. All eligible patients were enrolled in the study after informed consent. Treatment arm allocation was done as per treating surgeons' preference and since it was an observational study, investigators had no control over it. All the patients of DFU received conventional wound treatment which consisted of thorough surgical wound debridement followed by daily wound cleaning with desloughing (EUSOL) and antiseptic solution like povidone iodine and application of sterile bandage dressing. Surgical debridement is repeated as and when needed. Once wound turns healthy without any visible slough use of desloughing and irritant antiseptic agent was discontinued and wounds were dressed with emollient agents like paraffin tulle to avoid overgrowth of granulation tissue and sterile bandage. Wound care was performed by surgical resident doctor assisted by the faculty of Department of General Surgery.

Multimodal glycemic control plan with dietary modifications and oral hypoglycemic drugs, and insulin if needed was utilized with aggressive sugar monitoring for all patients.

In addition to above conventional management; study group also received topical ozone gas treatment as follows.

### Steps of Ozone Therapy

Day 1 of ozone therapy: wound is cleansed with saline and wound swab collected for microbial testing. Topical ozone gas therapy was given by bagging technique.

**Fig. 1:**
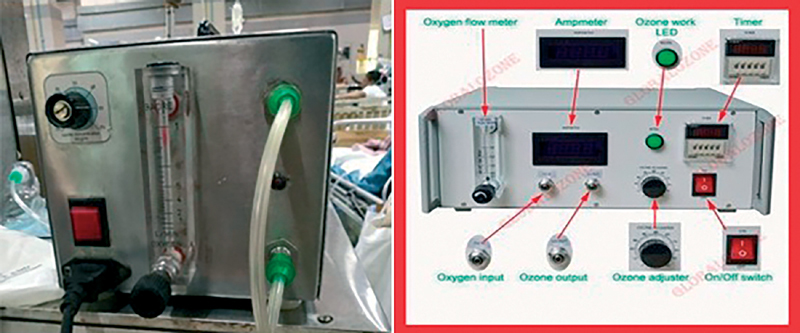
Ozone generator apparatus.

Ozone gas is generated by medical ozone gas generator ( Fig. 1 ). Pure oxygen is passed through a high voltage gradient in the generator (5–13 mV) which generates ozone according to this chemical reaction: 3O _2_ + 68.400 cal → 2O _3_ . 4 Ozone concentration in real time can be determined by a well standardized photometer or iodine titration test. 4

### Bagging Technique

**Fig. 2:**
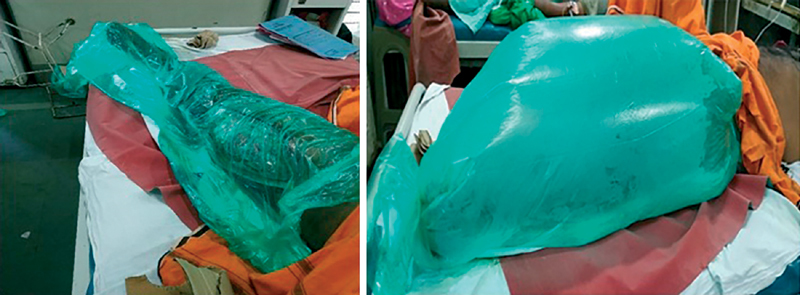
Bagging technique of ozone gas therapy to lower limb wound.

Wound is cleaned thoroughly with normal saline. A piece of saline soaked sterile gauze is placed over the wound to avoid direct exposure of wound to ozone. A bag is formed as per the size of the wound with use of sterile polythene sheet and adhesive sticking. Bag is placed over the wound and ozone gas outlet pipe is inserted in the bag. Mouth of this bag is sealed by roller bandage to avoid gas leakage ( Fig. 2 ). Ozone generator is then connected to oxygen source @1 L/min. Acrid smell is experienced when machine is switched on due to minor leak through bag which is unavoidable. Rate of ozone insufflation is adjusted between 35 and 45 µg/mL. Ozone insufflation is given for 5 minutes. Ozone machine is then switched off, outlet pipe taken out from the bag, and its mouth tightened. The inflated bag is kept for 1 hour exposure time for ozone to act on the wound. After 1 hour bag is removed, and sterile dressing done with conventional method.

Five such sessions of ozone therapy are given on alternate day.

Wound swab was taken on day 1 and every fourth day until microbial negativity and then weekly thereafter for all patients.

### Clinical Assessment of Wound

Wound assessment done on day—1, 4, 8, 14, and 21 days for both the groups and each time following outcome parameters were measured.

**Fig. 3:**
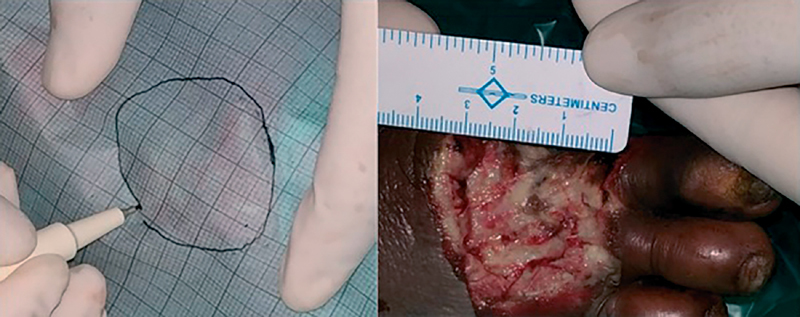
Ulcer surface area measurement with grid line paper.

1. *Wound size* : Sterile transparent polyethylene dressing placed over the wound which was already covered partially with a sterile gauze in such a way that edges of wound were exposed. A transparent acetate sheet with grid lines tracing is then placed over this sterile polyethylene transparent sheet through which underlying ulcer boundaries could be seen and marked on grid sheet ( Fig. 3 ). Surface area of big square on grid sheet was 1 cm ^2^ and that of smallest square was 1 mm ^2^ .Different color markers were used for every sequential reading. Surface area of the ulcer is calculated by counting the number of squares contained within the marked boundary of the ulcer tracing. Ulcer diameter was the longest diagonal of that ulcer on the tracing sheet.
2. *Character of exudate* : It was recorded by observing the kind of soakage of dressing material. Blood-stained dressing indicates serosanguinous exudate and semi-purulent or purulent exudate is seen by just pressing the wound or it's surrounding area.
  Grade 1: Serous for watery thin discharge.
  Grade 2: Serosanguinous for discharge stained with blood.
  Grade 3: Semi-purulent for thin turbid discharge.
  Grade 4: Purulent for presence of frank pus in the wound.
3. *Granulation tissue* : It is newly formed connective tissue and microscopic blood vessels that form on the floor of ulcer during healing process. Characteristics of healthy granulation tissue are light red or dark pink in color, moist, soft to touch, punctate bleeding on touch due to newly formed capillary loops, absence of slough, no purulent discharge, and painless. Proportion of ulcer area with healthy granulation tissue corresponds to the extent of healing. It was assessed by visual inspection and graded as below. Granulation tissue consisting of
  Score 1: <25% of area of the wound
  Score 2: 25 to 50% of area of the wound
  Score 3:50 to 75% of area of wound
  Score 4: >75% of area of wound.
4. *Edges of the wound* : Sloping edges suggest healing wound. It was assessed as % of the wound edge that is sloping.

➢ <25% ➢ 25–50% ➢ 50–75% ➢ >75%.

Patients were followed up during the hospital stay or within 30 days of admission whichever was later to know if he/she required a revision (re-debridement and amputations) surgery and to know the outcome of the patient.

All the relevant data were entered in predesigned case record form and compared between two groups for each of the outcome parameter to find out statistical significance.

## Results

In our study we had total of 162 patients, 81 each in C and O + C therapy group.

*Wound size* : In our study *n* = 162, median values of wound surface and maximum wound diameter of C ( *n* = 81) and O + C ( *n* = 81) are calculated on day 1, 4, 8, 14, and 21 ( Table 1 ).

**Table 1:** Association of mean decrease in wound surface area and maximum wound diameter with C and O + C therapy

Days	Mean surface area (C) therapy, cm ^2^	Mean surface area (O + C) therapy, cm ^2^	Mean of largest ulcer diameter (C) therapy, cm	Mean largest ulcer diameter (O + C) therapy, cm
Day 1	17.43	17.87	5.48	5.52
Day 4	17.06	17.16	5.37	5.39
Day 8	16.58	16.20	5.21	5.20
Day 14	15.85	14.77	4.99	4.93
Day 21	15	13.10	4.73	4.62

**Fig. 4:**
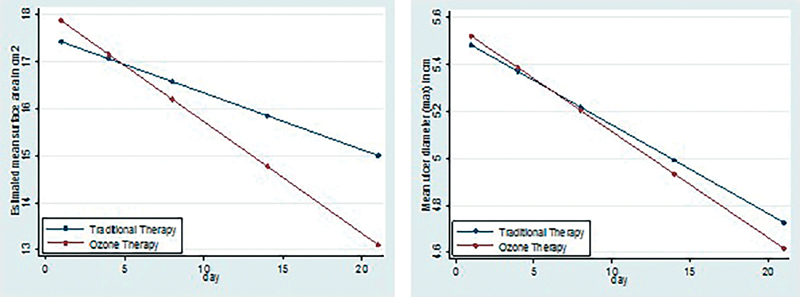
Association of mean decrease in wound surface area and wound diameter with Conventional (C) and (O + C) therapy.

Thus, the change or reduction in mean values of wound surface area and largest wound diameter have significant difference between two groups. If we plot a graph comparing these values of two groups, it shows significant change as shown in Fig. 4 .

On applying, generalized estimated equation for continuous data with interaction term to see the independent association of therapy with ulcer surface area and maximum ulcer diameter, significant interaction term suggest that intervention differences related to ulcer surface area are significantly changing over time with *p* -value <0.05. Trajectories of ulcer surface areas (change in ulcer surface area over the period of time) and that of largest ulcer diameter for two interventions were significantly different ( Table 1 and Fig. 4 ).

Thus, percent change in ulcer surface area after 21 days was higher 32.37% in O + C group compared with O alone group. And this change or reduction was statistically significant ( *p* = 0.01)

Similarly mean reduction in largest ulcer diameter after 21 days was higher for O + C group (18.62%) compared with C group (14.02%) which was statistically significant ( *p* = 0.01) ( Table 2 )

**Table 2:** Percent change in wound surface area and maximum wound diameter between day 1 and 21 in C and O + C group

	C ( *n* = 81)	O + C ( *n* = 81)	Unpaired *t* -test
% Change in surface area, mean (SD)	17.15 (7.48)	32.37 (11.49)	*t* -Value = − 9.92; *p* -Value = 0.01;
% Change in largest diameter, mean (SD)	14.02 (4.68)	18.62 (7.53)	*t* -Value = − 4.66; *p* -Value = 0.01;

2. *Association of character of wound exudate with C and O* *+* *C group* : Character of exudate can be purulent, seropurulent, serosanguinous, or serous and they were respectively given scores as 4, 3, 2, and 1.

**Fig. 5:**
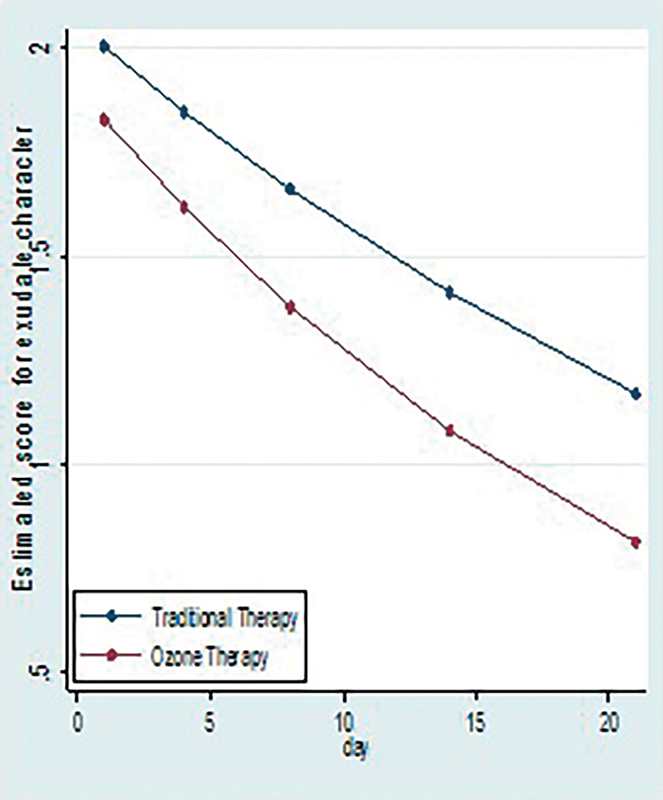
Comparison of median scores of characters of exudate between two study groups.

When we apply generalized estimated equation for count data with interaction term to see the independent association of therapy with character of exudate, there is significant interaction term which suggests that intervention differences related to character of exudate are significantly changing over time with *p* -value <0.05 ( Fig. 5 and Table 3 ).

**Table 3:** Comparison of median scores of characters of exudate between two study groups

Therapy		
Character of exudate, median (IQR)	C	O + C
Day 1	2 (2, 2)	2 (2, 2)
Day 4	2 (2, 2)	2 (1, 2)
Day 8	1 (1, 2)	1 (1, 1)
Day 14	1 (1, 1)	1 (1, 1)
Day 21	1 (1, 2)	1 (1, 1)

3. Association of rate of formation of healing granulation tissue with C and O + C therapy: To assess the rate of formation of healing granulation tissue scores were given as follow: <25% = 1, 25–50% = 2, 50–75% = 3, >75% = 4.

**Fig. 6:**
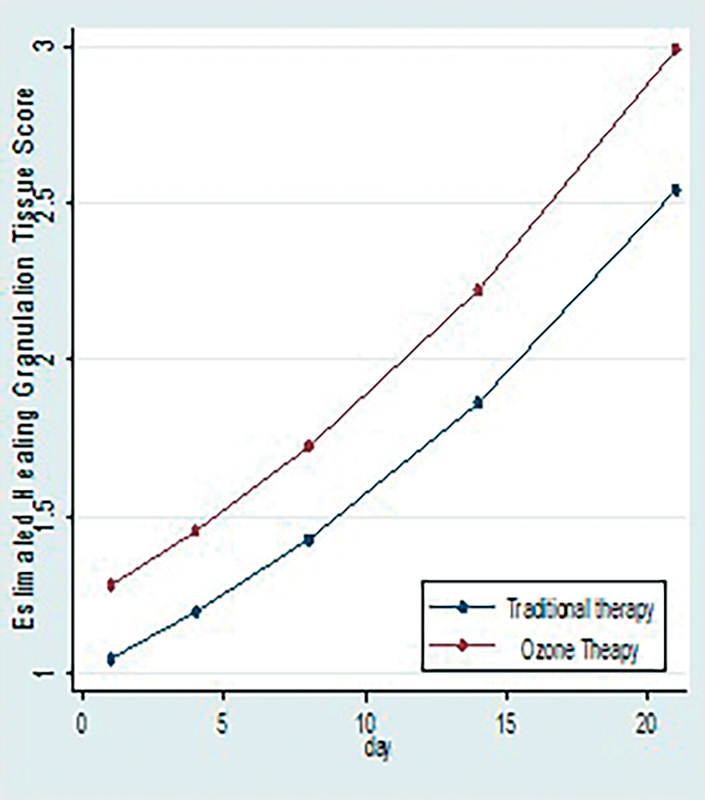
Comparison of scores of rates of formation of healing granulation tissue in two study groups.

When we apply generalized estimated equation for count data with interaction term to see the independent association of therapy with rate of formation of healing granulation tissue, there is nonsignificant interaction term which suggests that intervention differences related to healing granulation tissue are not significantly changing over time with *p* -value >0.05. Trajectories of healing granulation tissue (change in healing granulation tissue over the period of time) for two interventions were not significantly different ( Fig. 6 and Table 4 )

**Table 4:** Comparison of scores of rates of formation of healing granulation tissue in two study groups

Healing granulation tissue, median (IQR)	C	O + C
Day 1	1 (1, 1)	1 (1, 1)
Day 4	1 (1, 1)	1 (1, 1)
Day 8	2 (1, 2)	2 (2, 2)
Day 14	2 (2, 2)	2 (2, 2)
Day 21	2 (2, 3)	3 (3, 3)

(So, we will remove the interaction term from the model and rerun it). At any given time point, healing granulation tissue score is estimated to be 20% more for patients receiving O + C therapy compared with patient receiving C therapy. This difference was statistically significant.]

4. Association of rate of formation of healing wound edges with C and O + C therapy: To assess the rate of formation of healing wound edges scores were given as follow: <25% = 1, 25–50% = 2, 50–75% = 3, >75% = 4.

**Fig. 7:**
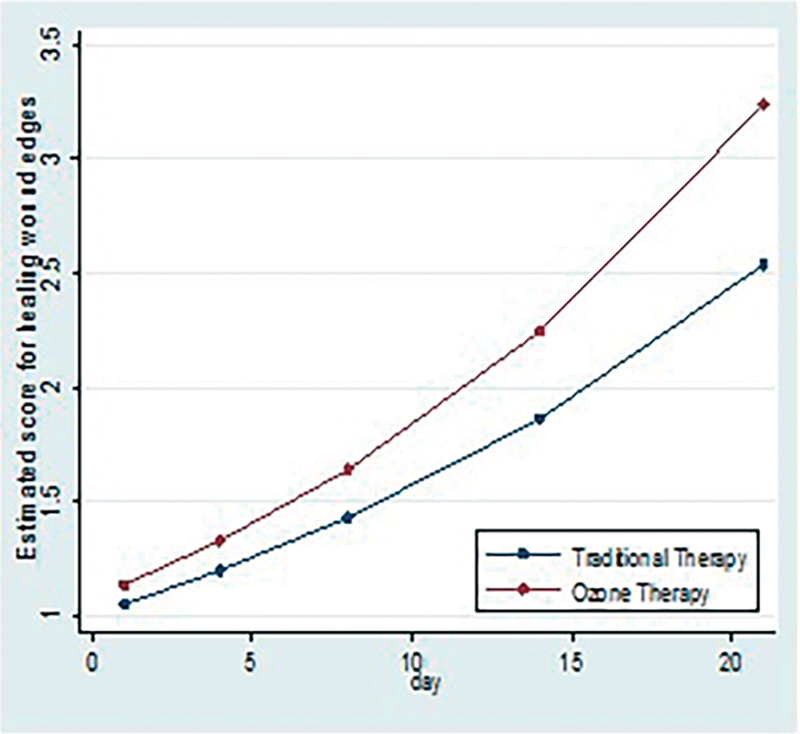
Comparison of scores of rates of formation of healing wound edges in two study group.

On day 21, healing wound edges score was estimated to be 27% more for patients receiving O + C therapy compared with patient receiving C treatment. This difference was statistically significant. When we apply generalized estimated equation for count data with interaction term to see the independent association of therapy with healing wound edges, there is significant interaction term, which suggests that intervention differences related to healing wound edges are significantly changing over time with *p* -value <0.05 ( Fig. 7 and Table 5 )

**Table 5:** Comparison of scores of rates of formation of healing wound edges in two study group

Healing wound edges, median (IQR)	C	O + C
Day 1	1 (1, 1)	1 (1, 1)
Day 4	1 (1, 1)	1 (1, 1)
Day 8	2 (1, 2)	2 (2, 2)
Day 14	2 (2, 2)	3 (2, 3)
Day 21	2 (2, 3)	3 (3, 3)

5. Adverse effects of ozone: Acrid odor: To assess the noxious nature of odor of ozone gas we used the Likert scale with score of 0 to 5.

**Fig. 8:**
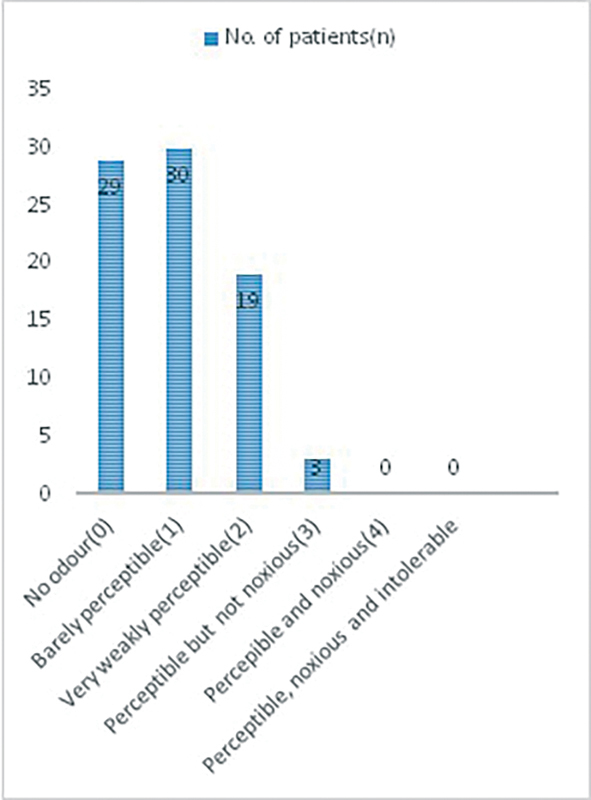
Likert scale and distribution of patients having different degree of odor intensity.

Ozone has acrid odor as an adverse effect but it is not noxious and is well tolerated by patients as shown in Fig. 8 and Table 6 .

**Table 6:** Likert scale and distribution of patients having different degree of odor intensity

Acrid odor for ozone therapy	Number (%)
0—No odor	29 (35.8)
1—Barely perceptible	30 (37)
2—Very weakly perceptible	19 (23.46)
3—Perceptible but not noxious	3 (3.7)
4—Perceptible and noxious	0
5—Perceptible, noxious, and intolerable	0

**Fig. 9:**
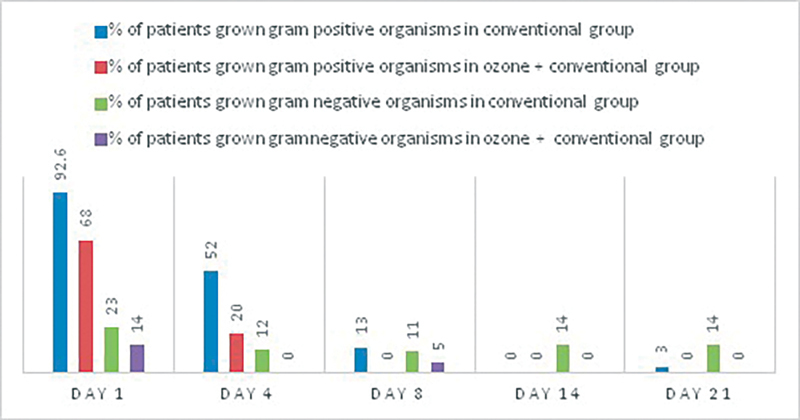
Comparison of percentages of patients whose ulcers grew gram-positive and gram-negative organisms in both the study groups.

6. Association of rate of microbial negativity with C and O + C therapy: Wound culture and antimicrobial sensitivity testing done on day 1, 4, 8, 14, and 21 for patients of both the groups. Fig. 9 shows faster rate of microbial negativity reaching 0% by day 8 for gram-positive and day 4 for gram-negative organisms in O + C group.

**Fig. 10:**
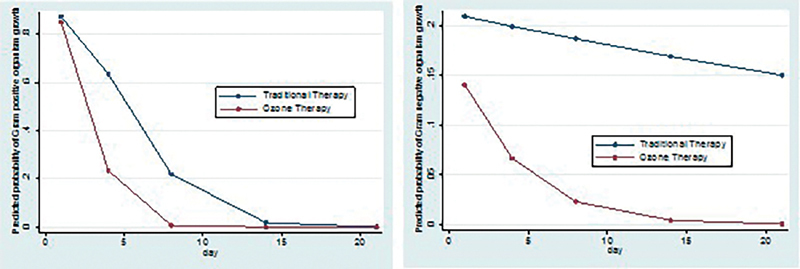
Predicted probability of gram-positive and gram-negative microorganisms over 21 days in both the study group.

To assess the significance statistically we apply generalized estimated equation for binary data with interaction term to see the independent association of therapy with gram-positive organism growth; there is significant interaction term which suggests that intervention differences related to the presence of gram-positive organism growth are significantly changing over time with *p* -value <0.05. Trajectories of the presence of gram-positive organism growth (change in presence of gram-positive organism growth over the period of time) for two interventions were significantly different ( Fig. 10 ).

**Fig. 11:**
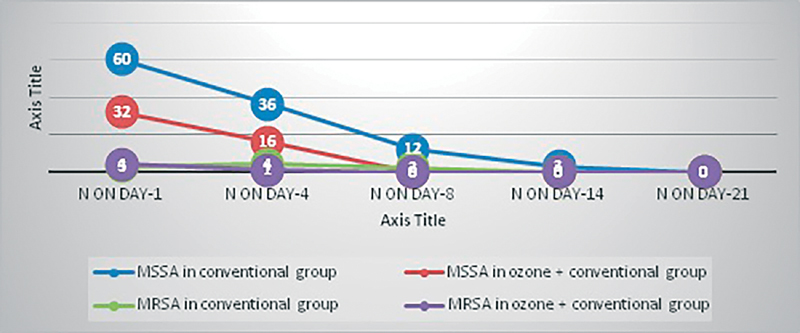
Trend of MSSA and MRSA in C and O + C group over 21 days. MRSA, methicillin-resistant *Staphylococcus aureus* ; MSSA, methicillin-susceptible *Staphylococcus aureus* .

**Fig. 12:**
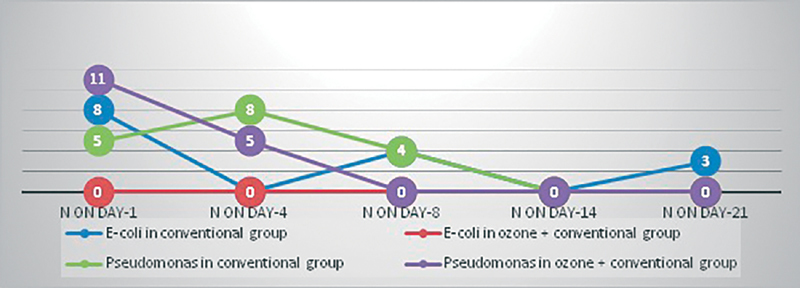
Trend of *E. coli* and *Pseudomonas* in C and O + C group over 21 days.

There is faster rate of microbial negativity with respect to methicillin-susceptible *Staphylococcus aureus* (MSSA), methicillin-resistant Staphylococcus aureus (MRSA), *Escherichia coli,* and *Pseudomonas* in O + C group than C group ( Figs. 11 and 12 ).

7. Association of hospital stay with C and O + C group: Since the median hospital stay is 9 days in O + C group compared with 13 days in C group and *p* -value 0.01 on application of Mann Whitney test, reduction in hospital stay is statistically significant for O + C group ( Table 7 )

**Table 7:** Comparison of hospital stay in two study groups

	C ( *n* = 81)	O + C ( *n* = 81)	Mann-Whitney U test
Hospital stay, median (IQR)	13 (11, 16)	9 (8, 9)	*z* -Value = 8.16; *p* -Value = 0.01

8. Association of revision (re-debridement and amputations) surgery required in C and O + C group: Patients were followed up for 30 days for outcomes of requirement of revision surgery and mortality. Table 8 shows that, 42% patients required revision surgery in C group while O + C group did not require revision surgery with *p* -value 0.01. This suggests statistically significant reduction in requirement of revision surgery in O + C group.

**Table 8:** Comparison of revision (re-debridement and amputations) surgery required in two study groups

		C ( *n* = 81), (%)	Ozone + C ( *n* = 81), (%)	Chi-square test
Revision surgery within 30 d	No	47 (58.0)	81 (100.0)	Chi-square value: 43.03; *p* -Value: 0.01;
	Yes	34 (42.0)	0 (0.0)	

9. Role of ozone gas therapy in the prevention of 30-day mortality: Since the Fischer exact test is showing *p* -value of 0.04 ( Table 9 ), this outcome parameter is statistically significant which suggests ozone therapy is associated with reduced mortality.

**Table 9:** Comparison of mortality within 30 d post-surgery

		C ( *n* = 81), (%)	Ozone + C ( *n* = 81), (%)	Chi-square test
Outcome	Alive	77 (95.1)	81 (100.0)	Chi-square value: 4.1; *p* -Value by Fischer exact test: 0.04;
	Death	4 (4.9)	0 (0.0)	

## Discussion

### Rate of Wound Healing

In double blind randomized control trial (RCT) by Wainstein et al in 2011 5 with O + C, among the 34 subjects who completed the study per protocol (PP) (16 in the O + C group, 18 in the placebo group), a significantly higher rate of complete wound closure was observed in the O + C group (81 vs. 44%, *p* = 0.03). Among PP patients with wound size ≤5 cm ^2^ , the rate of total wound closure was 100 versus 50% in the sham treatment group ( *p* = 0.006). *p* -Value in this study ( *p* = 0.006) is more significant than our study ( *p* = 0.01); this might be because of increased sessions of ozone gas therapy.

In Zhang et al 3 (RCT) the effective wound healing rate of O + C group was significantly higher than that of control group (92 vs. 64%, *p* < 0.05). The wound size reduction was significantly more in O + C group than in control group ( *p* < 0.001). *p* -Value in this study ( *p* = 0.001) is more significant than our study ( *p* = 0.01) and again this might be because of increased sessions of ozone gas therapy.

Number of sessions ozone to be given and rate of wound healing might be related as seen in Wainstein et al and Zhang et al but large scale RCT is needed to prove this hypothesis.

Ozone is capable of reacting to wide range of organic and inorganic biological substances to cause oxidation. Some of these substrates are proteins, amino acids, and unsaturated fatty acids, which form part of the lipoprotein complexes of plasma and of the double layers of the cellular membranes. Ozone reacts with membrane phospholipid bilayer to form some elements like ozonides, aldehydes, peroxides, and hydrogen peroxide (H _2_ O _2_ ). They interact with cellular DNA and cysteine residue and release second messengers, they activate enzymes, such as chemical and immune-response mediators in controlled manner to bring about therapeutic effect of ozone on healing. 6 Some of the other mechanisms of ozone promoting healing of ulcer are activation of aerobic processes by stimulation of Krebs cycle, broad spectrum antimicrobial property, antioxidant action, stimulation of growth factors, expression of adaptive inflammatory response, induction of synthesis of interleukins and leukotrienes, secretion of vasodilators like nitric oxide, and enhancement of immune system. 7 8 9 10

The immunological action of ozone on the blood is directed, fundamentally, to the monocytes and T lymphocytes, which once induced, releases small quantities of practically all the cytokines in an endogenous and controlled manner. This regulation is affected by the ozone which acts as an enhancer of the immunological system by activating neutrophils and stimulating synthesis of some of these cytokines. 11 12

Certain transcription factors like NFK-β intervene in the regulation which favor the transcription and transduction processes at DNA level and lead to the upregulation or suppression of synthesis of either pro-inflammatory or anti-inflammatory cytokines ( Fig. 4 ). 13

### Character of Ulcer Exudate

The character of ulcer exudate changes from purulent to seropurulent after debridement and as ulcer heals it converts to serosanguinous then finally to serous. This change is faster in O + C group compared with C group ( *p* <0.05, generalized estimation equation). Hence character of ulcer exudate is one of the good assessment parameter for healing of ulcers. No studies are present in the current scenario to study this parameter.

### Rate of Formation of Healing Granulation Tissue

Healing granulation tissue indicates recovery of ulcer from infection. At any given time point, in our study, healing granulation tissue score is estimated to be 20% more for patients receiving O + C therapy compared with patient receiving C therapy. This difference was statistically significant. Hence ozone might help in induction of faster rate of formation of healing granulation tissue. No studies present in current scenario to study this parameter.

### Rate of Formation of Healing Wound Edges

Like healing granulation tissue, healing wound edges along with disappearance of signs of inflammation in surrounding skin area indicate early recovery of the ulcer. Presence of fibrotic, punched out, rolled out, or undermined ulcer edges indicate chronicity of ulcer. These are signs of nonhealing of ulcer. Slopping ulcer edges are considered healthy.

On day 21, healing wound edges score was estimated to be 27% more for patients receiving O + C therapy ( *p* < 0.05 by generalized estimation equation) compared with patient receiving C treatment. This difference was statistically significant. And disappearance of signs of inflammation in surrounding skin was much faster in O + C group. Hence ozone might help in disappearance of signs of inflammation and faster induction of healing of wound edges. This might be because of immune modulation function of ozone.

### Adverse Effects of Ozone

Ozone is not a drug and as such it does not cause side effects, does not cause allergic reactions, and in general has no reported interactions with other drugs. Ozone in general is well tolerated by the patients. But in excessive doses few patients feel sensation of heaviness. It is for short duration and is resolved spontaneously. The use of plastic bags permeable to ozone leads to discomfort such as headaches. If the proper material is not used, the ozone reacts with the plastic material and introduces toxic compounds in the blood which are responsible for the adverse effects described. 14 In summary, the side effects are related to high doses of ozone, inappropriate use of materials, and thus are easily preventable.

### Rate of Microbial Negativity

Early microbial negativity indicates potency and efficacy of the drug or intervention. Our study shows faster rate of microbial negativity, i.e., 100% by day 8 for gram-positive organisms and that by day 14 for gram-negative organisms in O + C group. While in C group microbial negativity was achieved on day 14 for gram-positive organisms and it was not achieved at all for gram-negative organisms (86% reduction).

Ozone is specifically effective against MSSA and MRSA (Gulmen et al and Song et al) 15 16 amongst gram-positive organisms while *E. coli* and *Pseudomonas* amongst gram-negative organisms. In a study by Fontes et al 17 MRSA was cleared on day 4 in O + C group while it persisted till day 8 in C group. *Pseudomonas* was cleared on day 8 in O + C group while it required 14 days to clear in C group.

In Song et al, 16 clinical efficacy and safety of topical ozone were evaluated in two cases with skin MRSA infection. The killing rates of ozonated oil for *S. aureus* and MRSA were greater when compared with the control oil group. Almost 100% of *S. aureus* were eliminated by ozonated oil in 5 minutes and MRSA in 15 minutes. In addition, 100% *S. aureus* and 100% MRSA were eliminated by ozonated water in 1 minute.

Gulmen et al 15 found that both the vancomycin and the ozone treatments caused significant reduction of bacterial counts in quantitative bacterial cultures. Histologic examination of tissue samples revealed significant reduction in severity of mediastinitis-related inflammation in vancomycin and vancomycin ozone groups compared with untreated contaminated group.

Fontes et al, 17 says that when selected ozone dose was applied to the following eight strains: *Escherichia coli* , oxacillin-resistant *Staphylococcus aureus* , oxacillin-susceptible *S. aureus* , vancomycin-resistant *Enterococcus faecalis* , extended-spectrum β-lactamase-producing *Klebsiella pneumoniae* , carbapenem-resistant *Acinetobacter baumannii* , *A. baumannii* susceptible only to carbapenems, and *Pseudomonas aeruginosa* susceptible to imipenem and meropenem. All isolates were completely inhibited by the ozone–oxygen mixture while growth occurred in the other 2 control groups.

#### Miscellaneous Effects of Ozone

Enas Mohammed Ali 18 studied the antifungal action of ozone gas therapy for DFU.

Spore viability of *C. albicans* was reduced by over 99.5% at 3 ppm ozone concentration after 180 minutes' exposure time. Prevention of mycelial growth in *A. flavus* was detected with 100% inhibition efficacy at 3 ppm ozone after 210 minutes. The study also determined the efficiency of ozonation in degrading mycotoxins produced by most dominant mycotoxigenic fungal species. The production of aflatoxins and trichothecene toxins was greatly inhibited at 3 ppm ozone after 180 minutes.

Wells et al 19 studied the antiviral effect of ozone on HIV-1 virus in vitro. Ozone was found to inactivate HIV-1 virions in a dose-dependent manner. Ozone treatment offers promise as a means to inactivate human retroviruses in human body fluids and blood product preparations.

#### Actions of Ozone 20

- It has high oxidizing capacity and acts on microbial wall, which acts on bacteria, viruses, and fungi; hence it has broad spectrum activity.
- It is effective even on highly resistant microbial flora.
- It improves the delivery of oxygen to the tissues.
- It improves the red blood cell metabolism, making the metabolism of glucose more efficient.
- It improves the metabolism of the fatty acids for the activation of antioxidant enzymes in charge of eliminating peroxides and free radicals.
- The principal metabolic effects attributed to ozone are:
  1. Increment of the use of glucose at the cellular level.
  2. It improves the protein metabolism.
  3. Direct effects on the unsaturated lipids, it oxidizes them and induces at the same time the repair mechanisms.
- Ozone has a dual action mechanism: analgesic and anti-inflammatory. These effects seem to be due to its way of acting on diverse targets:
  1. Decrease the production of mediators of the inflammation.
  2. The oxidation (inactivation) of metabolic mediators of pain.
  3. It clearly improves local blood microcirculation, with an improvement in the oxygen delivery to the tissues, essential for the regeneration of anatomical structures; the elimination of toxins and in general the resolution of the physiological disturbance that generated the pain.

### Reduction in Hospital Stay

Because of faster rate of ulcer healing and early microbial negativity, hospital stay of patients has significantly reduced in our study in O + C group. Hence ozone gas indirectly helps to decrease the overall cost expenditure for management of DFU. Rosul and Patskan studied RCT for DFU; there was significantly faster rate of wound healing, lipid peroxidation, reduction in hospital stay, greater antioxidant protection, and yielded significant decrease in microbial colonization of wounds. 21

### Requirement of Revision Surgeries (Re-debridement/Amputation)

Because of effective antimicrobial, antioxidant, and immune modulation property of ozone there was faster rate of microbial negativity and ulcer healing and decreased requirement of revision surgeries. Forty-two percent of patients required revision surgery in C group while O + C group did not require revision surgery with *p* -value 0.01 by Chi-square test. This suggests statistically significant reduction in requirement of revision surgery in O + C group. Similar study by Izadi et al 22 found similar results, i.e., there was decrease in chances of wound re-infections and amputations in O + C group compared with control group.

### Role in Mortality Prevention

There were no deaths observed in O + C group while percentage mortality in C group was 4.9% on 1 month follow-up. *p* -Value by Fischer exact test was found to be 0.04 which is statistically significant. Three deaths were seen in patients who required revision surgery and one of them had MRSA infection. These deaths were due to septic complications of diabetic foot and not due to other co-morbid conditions. As we discussed above, because of effective antimicrobial and immune modulation property of ozone there was faster rate of microbial negativity, ulcer healing, and decreased requirement of revision surgeries. Hence ozone has a role in prevention of both DFU morbidity and 30 day mortality.

### Limitations of This Study

1. Our study was nonrandomized.
2. Population was heterogenous with having different inherent wound healing capacity due to different age, gender, and co-morbid conditions leading to multiple confounding factors.
3. Smaller duration of follow-up (21 days) due to social or technical issues of the hospital.

### Strengths of This Study

1. Our study is prospective.
2. Accurate measurement of ulcer surface area and maximum diameter (with tracing paper with minimum surface area of 1 mm ^2^ ).
3. Since study population is heterogenous, results are applicable to wider range of population.
4. Various new parameters of DFU have been studied which have not studied previously, i.e., character of exudate, presence of ulcer odor, rate of formation of healing granulation tissue, and rate of formation of healing wound edges after application of ozone gas therapy.

## Conclusion

1. This study proves efficacy of topical ozone gas therapy and provides clinical evidence to support use of ozone. We recommend it for faster healing of DFU.
2. This study confirms antimicrobial potency of ozone gas therapy.
3. This study shows that dose of 40 µg/mL (range = 30–50 µg/mL) of topical ozone gas therapy is optimal for DFU. And ozone gas is well tolerated by patients at this concentration without any significant adverse effects.
4. We recommend using ozone gas therapy for reduction in hospital stay and reduction in requirement of revision (re-debridement and amputations) surgeries. It also has a role in reduction of 30 days mortality.
5. Thus, ozone therapy is very effective, cheap, simple, well tolerated, and easily reproducible but underutilized tool. It does not require huge infrastructure or complex equipment. Also, no recurring cost of consumables is involved making it a very suitable tool to promote wound healing in DFUs in resource-constraint regions.
